# Comparative analysis between Reverdin-Isham Osteotomy (RIO) and minimally invasive intramedullary nail device (MIIND) in association with AKIN osteotomy for Hallux valgus correction

**DOI:** 10.1186/s13018-025-05569-7

**Published:** 2025-02-20

**Authors:** Carlo Biz, Nicola Luigi Bragazzi, Anna Di Rita, Assunta Pozzuoli, Elisa Belluzzi, Maria Grazia Rodà, Pietro Ruggieri

**Affiliations:** 1 Department of Orthopedics and Orthopedic Oncology, Department of Surgery, Oncology and Gastroenterology DiSCOG, University-Hospital of Padova, Via Giustiniani 3, Padova, 35128 Italy; 2 Centre for Mechanics of Biological Materials, University of Padova, Padova, 35131 Italy; 3 Department of Mathematics and Statistics, Laboratory for Industrial and Applied Mathematics (LIAM), York University, Toronto, ON M3J 1P3 Canada; 4 Musculoskeletal Pathology and Oncology Laboratory, Department of Surgery, Oncology and Gastroenterology (DiSCOG), University of Padova, Via Giustiniani 3, Padova, 35128 Italy

**Keywords:** Hallux valgus, MIIND, Endolog, Sesamoids, RIO, Reverdin-Isham, Akin

## Abstract

**Background:**

Hallux valgus (HV) is a widespread condition that leads to discomfort in daily life. There are different surgical techniques for HV. This retrospective and comparative study aimed to compare the clinical and radiographic outcomes of the Reverdin-Isham osteotomy (RIO) and the Minimally Invasive Intramedullary Nail Device (MIIND) surgical techniques.

**Methods:**

One hundred ninety-six patients with mild-to-severe HV were enrolled and divided into two groups: 98 patients with mild-moderate HV and 98 with moderate-severe HV, treated with the RIO and MIIND techniques, respectively. Radiographic and clinical evaluations were assessed preoperatively at 3, 12 and 60 months after surgery. Radiologically, the Hallux Valgus Angle (HVA), Intermetatarsal Angle (IMA), Distal Metatarsal Articular Angle (DMAA) and Tibial Sesamoid Position (TSP) were evaluated. Clinically, the AOFAS hallux metatarsophalangeal-interphalangeal scale and the Numeric Rating Scale (NRS-11) for pain were assessed. A propensity score matching (PSM) model was implemented to compare the two techniques.

**Results:**

In the RIO group, the mean HVA correction from preoperative value to 60 months of follow-up was 8.69° (*p* < 0.0001), the mean IMA correction was 2.42° (*p* < 0.0001), and the mean DMAA correction was 0.09°. In the MIIND group, the mean HVA correction was 24.92° (*p* < 0.0001), the mean IMA correction was 8.75° (*p* < 0.0001), and the mean DMAA correction was 6.28° (*p* < 0.0001). The mean AOFAS score improved over time, and NRS-11 decreased in both groups. After PSM model application, the variables that impacted the allocation to RIO or MIIND techniques were age, preoperative HVA values and HV severity.

**Conclusion:**

Our study demonstrates the efficacy of RIO for mild-moderate HV and MIIND for moderate-severe HV. Radiographic and clinical outcomes improved in both groups, but older patients with higher HVA and severe HV should be treated with the MIIND technique to achieve satisfactory outcomes.

**Level of evidence:**

III, retrospective cohort study.

**Supplementary Information:**

The online version contains supplementary material available at 10.1186/s13018-025-05569-7.

## Introduction

Hallux valgus is a common forefoot deformity that causes pain, discomfort and difficulty walking, leading to decreased quality of life. The estimated prevalence of this condition is 19% in the general population, but it increases to 22.7% in individuals aged 60 or above, and it is more frequent in women (23.74% vs. 11.43% in males) [1]. The aetiology is multifactorial, involving intrinsic and extrinsic causes, even if not totally understood [2]. It is described as the progressive abduction and pronation of the first phalanx, adduction, pronation and elevation of the first metatarsal bone (MB), the lateral capsular retraction of the first metatarsophalangeal joint and dislocation of the sesamoids [3].

Surgical correction is the standard treatment of symptomatic HV and seems to be more effective than nonoperative methods [4]. More than 400 different surgical techniques have been described for HV correction, from open traditional procedures to percutaneous ones, consisting of various types of osteotomies at different levels of the first MB [5–11].

Minimally invasive surgery (MIS) and percutaneous techniques are becoming popular because of the good clinical and radiographic outcomes, smaller scars, lower postoperative pain, immediate weight bearing and shorter recovery [12–16].

Among percutaneous techniques, the Reverdin-Isham osteotomy (RIO), in combination with the Akin osteotomy, is performed with lateral soft tissue release and without internal fixation to correct mild-to-moderate deformity [17, 18].

The Minimally Invasive Intramedullary Nail Device (MIIND) consists of a curvilinear cylindrical titanium body and a blade producing a progressive lateral displacement of the first metatarsal head (MTH). It is preferably indicated to correct moderate-to-severe HV, allowing multiplanar correction of the deformity and the anatomic reduction of the sesamoids without performing a lateral release [19].

Only a few trials have compared percutaneous *versus* minimally invasive procedures, reporting no differences [4, 20–22]. However, these studies had small sample sizes and relatively short follow-ups.

This study aimed to compare the clinical and radiographic outcomes at long-term follow-up of patients surgically treated for painful HV using the RIO and the MIIND in association with Akin osteotomy.

## Materials and methods

### Study design

A retrospective, comparative, observational, single-centre cohort study of consecutive patients diagnosed with mild-to-severe HV was performed. After providing written informed consent, patients were enrolled from January 2014 to December 2018.

The Local Ethics Committee approved the study (4064/AO/17) that was carried out in accordance with the ethical standards from the Declaration of Helsinki, revised in 2024.

The inclusion criteria were as follows: patients between 18 and 80 years old with a diagnosis of HV with constant pain in the area of the first MTH not extending to other metatarsals, having particular discomfort while wearing shoes, and undergoing unilateral RIO or MIIND procedure.

The exclusion criteria were as follows: previous foot surgery or trauma, vascular insufficiency, diabetes mellitus, foot neuropathy, rheumatologic diseases, hallux rigidus, generalised joint laxity or hypermobility of the first ray, additional procedures on the lateral rays during the same operation and a follow-up less than 60 months [23].

Patients were categorised based on the Mann and Coughlin classification [24] for HV correction and underwent RIO or MIIND based on the severity of HV: (1) RIO + Akin for mild-moderate HV and (2) MIIND + Akin for moderate-severe HV. The same surgeon performed both techniques, leveraging his extensive experience with them, spanning nearly two decades. The choice of technique was guided by the outcomes and criteria established in previous case series. RIO was selected for mild-to-moderate HV correction, while MIIND was preferred for cases of moderate-to-severe deformity [19, 25, 26].

### Surgical techniques

Each operative technique included prophylactic antibiotic therapy before surgery and thromboembolic prophylaxis the same evening and for 30 days. Anaesthesia consisted of conscious sedation in association with a regional ankle block of superficial and deep nerves [25]. Both operative procedures were completed by Akin percutaneous osteotomy performed medially at the base of the proximal phalanx with medial base and lateral cortex preservation.

### RIO surgical technique

The RIO was performed as described by De Prado through two different skin incisions and under fluoroscopy control as described by Biz et al. [18, 26]. Through the first incision on the medial side of the first MTH, the exostosectomy and the distal osteotomy were performed without cutting the lateral cortex. Then, a wedge burr was used to create a wedge with a medially oriented base. At the point of closing the wedge, osteoclasis of the preserved lateral cortex was achieved, modifying the orientation of the articular surface, normalising the distal metatarsal articular angle (DMAA) value, and adding intrinsic stability to the osteotomy by producing contact of the trabecular bone. A scalpel was introduced through a second skin incision on the first metatarsal space to perform lateral soft tissue release and lateral capsulotomy. Finally, a bandage was applied to maintain the correction.

### MIIND surgical technique

The MIIND technique was conducted by providing a 3-cm dorsal-medial longitudinal incision centred on the exostosis of the first MB [19, 27]. A linear osteotomy at the proximal level of the metatarsal neck followed the bunionectomy. Correction of the DMAA and subluxation of the sesamoids were, then, achieved by inserting the trial nail device into the medullary cavity, with progressive lateral displacement of the MTH and its simultaneous derotation. Then, the Endolog, a curved intramedullary titanium nail (available in 3 different lengths and curvatures), was implanted and fixed to the MTH with a screw to provide angular stability [27].

The medial angle of the metatarsal neck was regulated with a micro-saw to prevent conflict of the bone with the soft tissues and skin. Finally, a compression dressing and tape were applied with the hallux slightly hypercorrect.

### Postoperative protocol

Postoperative treatment was standardised for both groups as previously described [19, 26, 27]. Soft dressings were applied and, after suture removal at two weeks, a postoperative bandage was reduced to allow full movement of the first metatarsophalangeal joint. Starting the evening after surgery, patients could walk as much as they tolerated using a rigid, flat-soled orthopaedic shoe for 30 days. Patients were instructed to wear an interdigital silicone orthoses spacer between the first and second toe for one month to help maintain the correct position of the first ray until the osteotomy fully consolidated.

### Patient assessment

Baseline characteristics of the patients were collected including age, gender, body mass index (BMI), smoking habits, side involved, use of narrow-tip shoes and/or high heels and family history of HV.

### Radiological and clinical evaluation

Radiographic and clinical follow-up assessments were performed at baseline, 3, 12 months after surgery and at the last follow-up of 60 months by two orthopaedic surgeons. Intraclass Correlation Coefficients (ICCs) for continuous variables were used to quantify the agreement levels. Intra-reader and inter-reader reliability were found to be good (> 0.80) for all measurements.

Radiological outcomes were evaluated using the MedStation program (Version 4.9).

Hallux valgus angle (HVA), intermetatarsal angle (IMA), distal metatarsal articular angle (DMAA), and the tibial sesamoid position (TSP) were measured and categorised with regard to deformity severity (Additional File 1) [28–30].

The 100-point hallux metatarsophalangeal-interphalangeal scale by the American Orthopaedic Foot and Ankle Society (AOFAS) was used to assess clinical outcomes [31]. Both preoperatively and at the last follow-up, pain was evaluated using the Numeric Rating Scale (NRS-11) [32], ranging from 0 (no pain) to 10 points (worst pain); only patients reporting NRS-11 ≥ 5 were operated.

Patient satisfaction was evaluated using the Visual Analogue Scale (VAS) for satisfaction, ranging from 0 (not satisfied) to 10 points (excellent result) [33]. Clinical complications were collected.

### Statistical analysis

An independent statistician from another institution conducted statistical analysis. Continuous data were presented as means and standard deviations, while categorical data were expressed as percentages where appropriate.

To compare the two techniques, a propensity score matching (PSM) model was implemented, which is a quasi-experimental, causal inference method in which subsets of the initial populations sharing similar and comparable characteristics are artificially created, removing or at least reducing the effects of biases and confounding due to non-random treatment allocation and treatment selection influenced by the subjects’ features. In this way, the impact of the two interventions and their outcomes could be estimated by accounting for differences in baseline characteristics, and other confounders and could be compared directly [34].

**Fig. 1:**
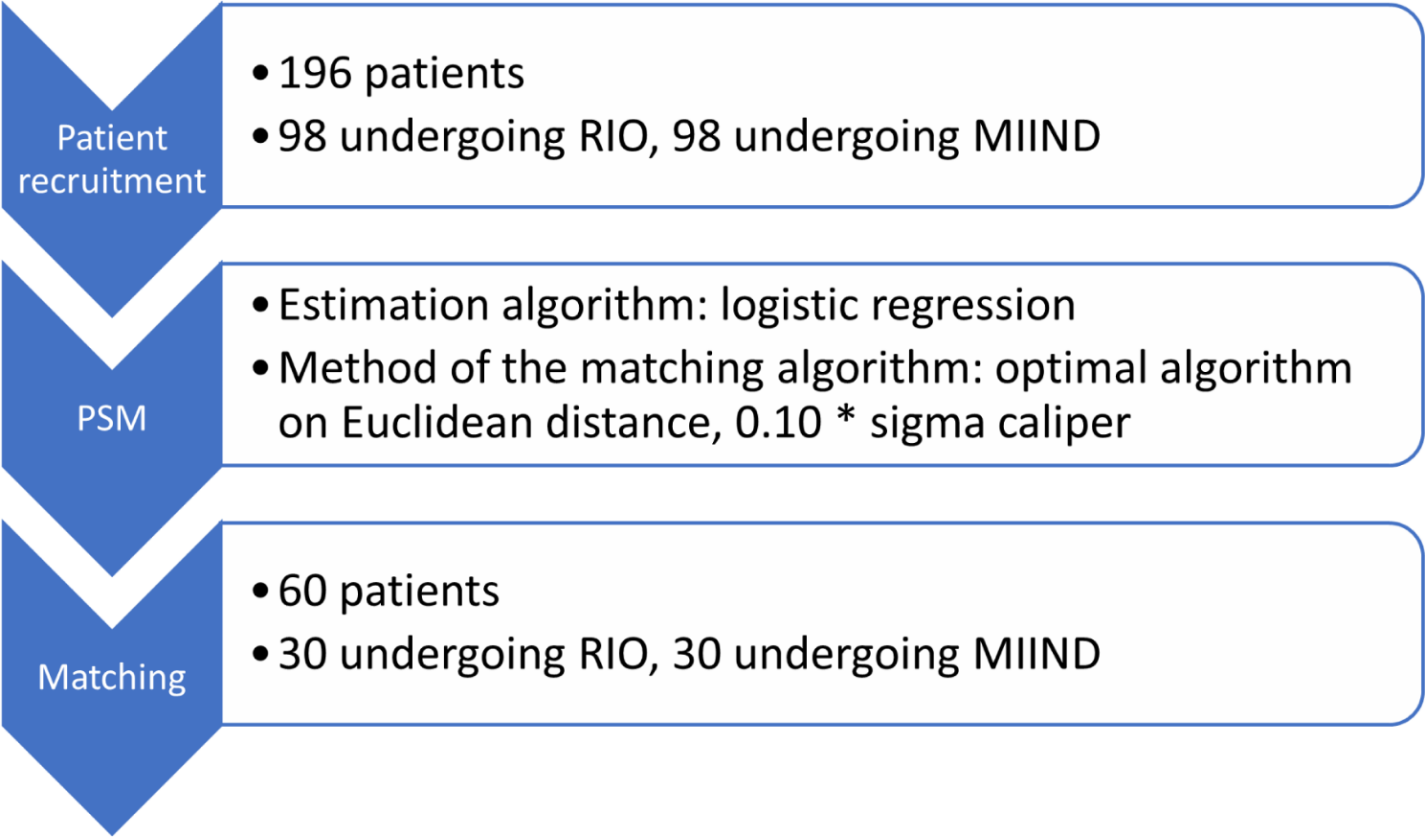
Flowchart of the PSM algorithm

**Fig. 2:**
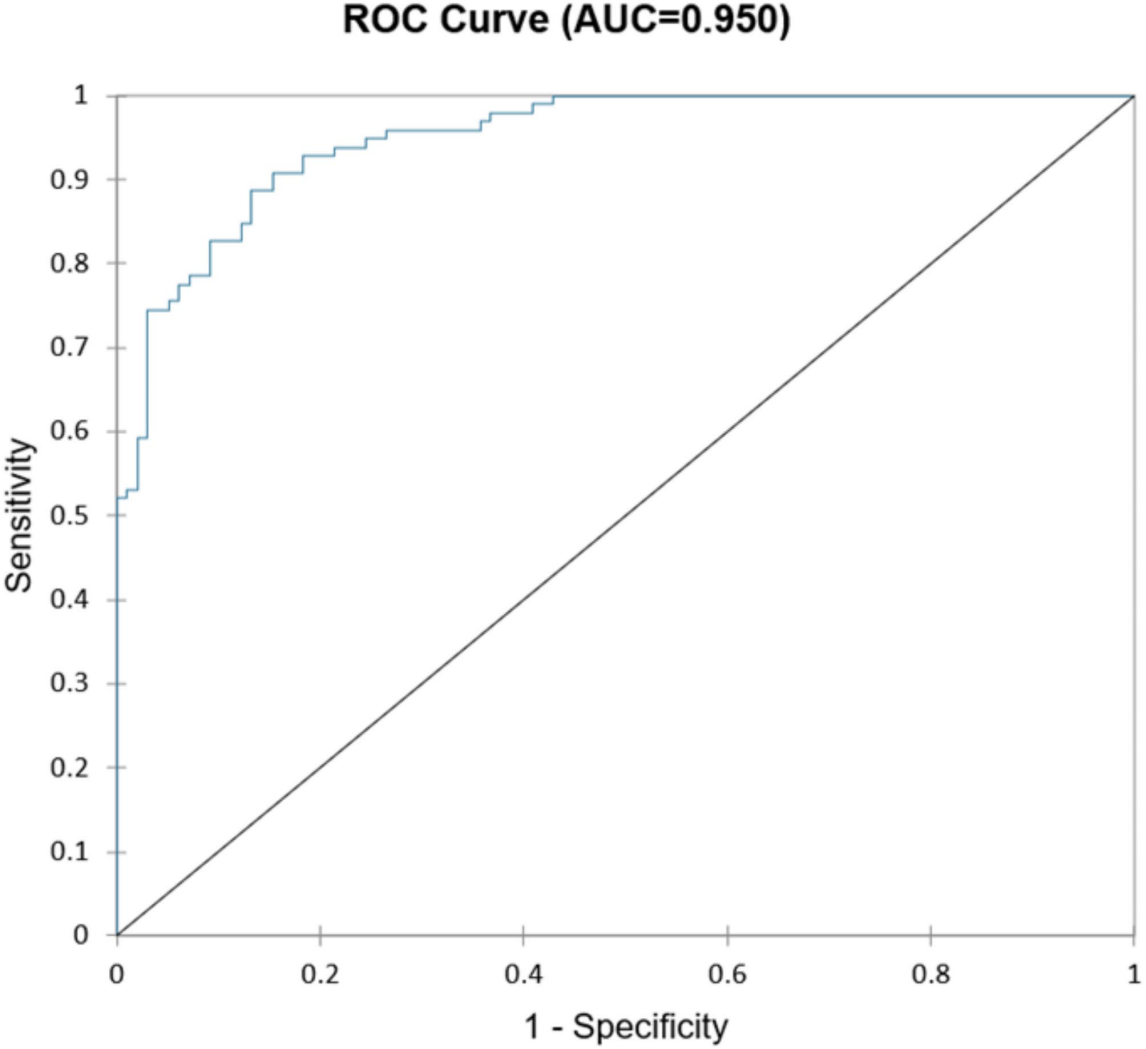
ROC curve of the logistic regression model that has informed the PSM algorithm

PSM was carried out employing the commercial software XLSTAT (version for Windows OS, Lumivero) using an optimal algorithm based on the Euclidean distance, with a one-to-one match in the number of matches, and 0.10 * sigma as caliper size option (Fig. 1) [35]. The propensity score was estimated using a logistic regression model in which the surgical treatment (RIO vs. MIIND) status was regressed on observed characteristics (covariates and factors). The impact of each variable was visually inspected by plotting their standardized coefficients. The quality of the model was confirmed by several indicators, including − 2 Log (Likelihood), R² according to McFadden, Cox and Snell, and Nagelkerke, Akaike Information Criterion (AIC), Schwarz Bayesian Information Criterion (SBC), and the area-under-the-curve (AUC) from the Receiver Operating Characteristic (ROC) analysis. The quality of the model was found to be excellent (Fig. 2).

**Fig. 3:**
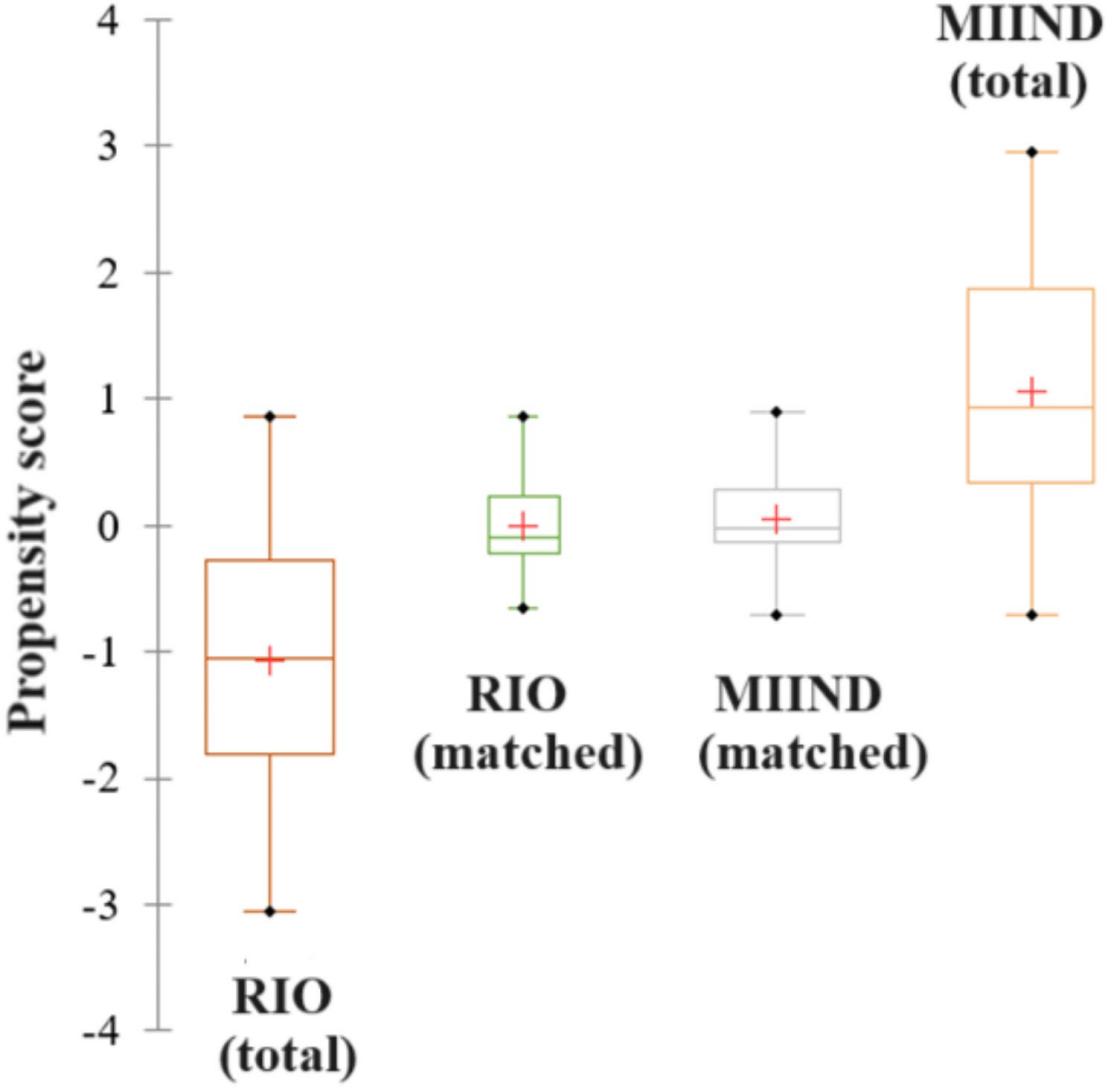
Boxplots showing the result of the PSM

The matching percentage was 31%, resulting in thirty subjects per group. Diagnostics, including balancing boxplots, were visually inspected to verify the effects of the matching operation on several parameters of the distribution of the propensity score within each group [36]. Distributions were found to be comparable after the matching operation, differently from before (Fig. 3). Differences between the two matched groups were computed using Student’s t-test for paired samples (or its non-parametric version) [37].

Given the relatively low matching percentage, a sensitivity analysis was conducted using an ordinary least-squares regression mixed model for repeated measures data applied to the entire dataset, which yielded comparable results (Additional File 2).

## Results

### Patient data

**Fig. 4:**
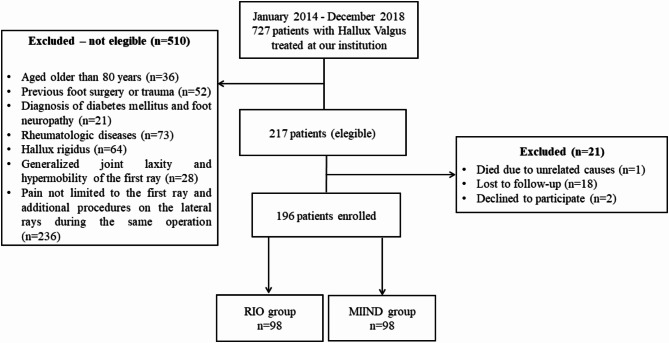
Flowchart of cohort selection

During the analysis period, 727 patients were operated on for painful HV using the two procedures. After applying inclusion and exclusion criteria, 217 patients were recruited, of which 21 were excluded. Hence, 196 patients were eligible and divided into two groups, according to the technique used: 98 patients by RIO and 98 by MIIND (Fig. 4).

Demographic characteristics of the entire cohort, divided according to the type of surgery, are reported in Table 1. The last follow-up was 60 months.

**Table 1:** Main demographic, clinical, and radiological features of the initial population (*n* = 196) and divided according to the type of surgery

Parameter	Initial population (*n* = 196)	RIO (*n* = 98)	MIIND (*n* = 98)
Age	55.85 ± 14.39	49.55 ± 14.44	62.14 ± 11.29
Sex/gender			
Male	28 (14.3%)	12 (12.2%)	16 (16.3%)
Female	168 (85.7%)	86 (87.8%)	82 (83.7%)
BMI	26.21 ± 3.69	26.23 ± 3.77	26.20 ± 3.62
Smoking			
No	144 (73.5%)	64 (65.3%)	80 (81.6%)
Yes	52 (26.5%)	34 (34.7%)	18 (18.4%)
Side			
Right	99 (50.5%)	51 (52.0%)	48 (49.0%)
Left	97 (49.5%)	47 (48.0%)	50 (51.0%)
Hallux valgus			
Grade 1	41 (20.9%)	41 (41.8%)	0 (0.0%)
Grade 2	115 (58.7%)	57 (58.2%)	58 (59.2%)
Grade 3	40 (20.4%)	0 (0.0%)	40 (40.8%)
HVA (°) preoperative	29.39 ± 9.97	23.36 ± 5.87	35.42 ± 9.57
IMA (°) preoperative	13.26 ± 3.151	11.95 ± 2.60	14.57 ± 3.12
DMAA (°) preoperative	11.97 ± 6.20	9.88 ± 5.68	14.05 ± 6.02
HVA (°) 3 months	11.87 ± 6.95	13.94 ± 6.48	9.81 ± 6.83
IMA (°) 3 months	8.04 ± 3.45	9.74 ± 2.61	6.33 ± 3.36
DMAA (°) 3 months	8.66 ± 6.36	9.88 ± 7.28	7.43 ± 5.01
HVA (°) 12 months	12.26 ± 7.41	13.99 ± 6.22	10.53 ± 8.09
IMA (°) 12 months	7.65 ± 3.32	9.43 ± 2.52	5.87 ± 3.07
DMAA (°) 12 months	8.26 ± 5.94	8.87 ± 6.41	7.66 ± 5.38
HVA (°) 60 months	12.59 ± 7.75	14.67 ± 6.43	10.50 ± 8.41
IMA (°) 60 months	7.67 ± 3.56	9.53 ± 2.83	5.82 ± 3.24
DMAA (°) 60 months	8.78 ± 7.04	9.79 ± 7.88	7.77 ± 5.96
Sesamoids preoperative	2	1	3
Sesamoids 3 months	1	1	0
Sesamoids 12 months	1	1	0
Sesamoids 60 months	1	1	0
AOFAS preoperative	50.29 ± 7.47	52.63 ± 6.53	47.95 ± 7.64
AOFAS 3 months	70.60 ± 7.67	71.24 ± 7.89	69.95 ± 7.43
AOFAS 12 months	80.43 ± 10.01	79.29 ± 11.20	81.57 ± 8.55
AOFAS 60 months	86.52 ± 11.51	85.23 ± 13.02	87.80 ± 9.68
NRS11 preoperative	6.93 ± 1.30	6.41 ± 1.10	7.46 ± 1.28
NRS11 60 months	1.27 ± 1.37	1.23 ± 1.36	1.31 ± 1.38
VAS 60 months	7.15 ± 2.18	6.84 ± 2.19	7.46 ± 2.13
Use of narrow-tip shoes			
Yes	85 (43.4%)	54 (55.1%)	31 (31.6%)
No	111 (56.6%)	44 (44.9%)	67 (68.4%)
Use of high heels			
Yes	88 (44.9%)	57 (58.2%)	31 (31.6%)
No	108 (55.1%)	41 (41.8%)	67 (68.4%)
Use of safety shoes			
Yes	12 (6.1%)	6 (6.1%)	6 (6.1%)
No	184 (93.9%)	92 (93.9%)	92 (93.9%)
Positive family history			
Father	9 (4.6%)	4 (4.1%)	5 (5.1%)
Mother	65 (33.2%)	44 (44.9%)	21 (21.4%)

MIIND = Minimally Invasive Intramedullary Nail Device; RIO = Reverdin Ishan Osteotomy; BMI = Body Mass Index; HVA = hallux valgus angle; IMA = intermetatarsal angle; DMAA = distal metatarsal articular angle; AOFAS = American Orthopaedic Foot and Ankle Society score; NRS = Numeric Rating Scale for pain; VAS = visual analogic scale for satisfaction

Data are reported as mean ± standard deviation unless otherwise indicated

### Radiographic outcomes

#### RIO group

**Fig. 5:**
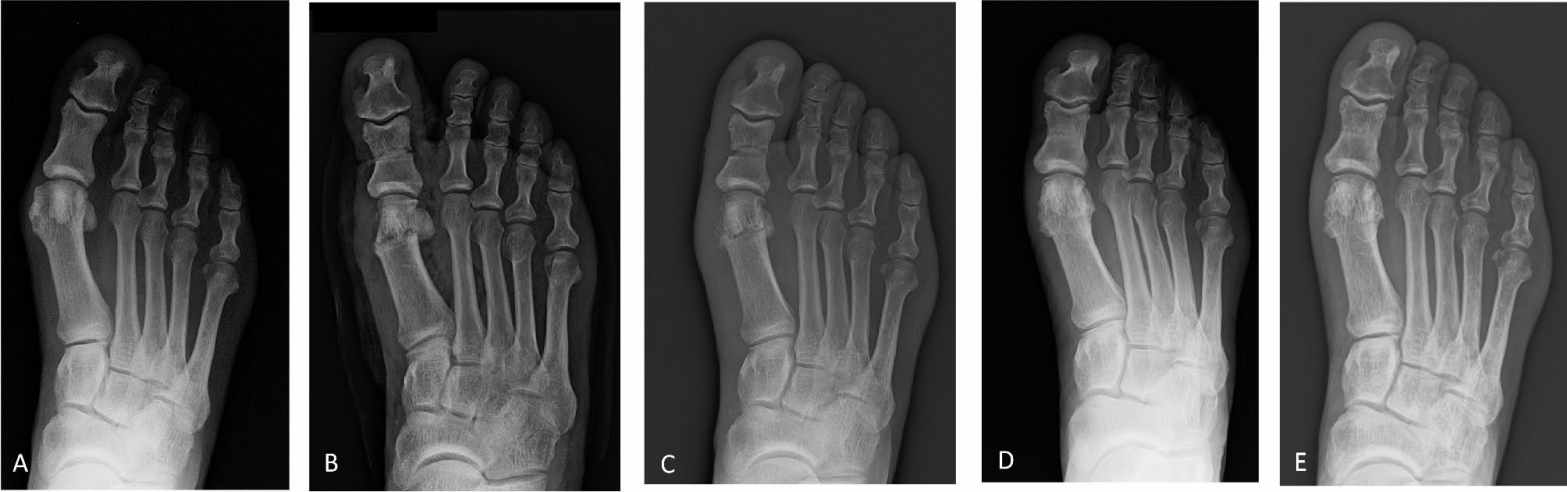
Case 1: A 69-year-old man having undergone the Reverdin-Isham and Akin osteotomies for moderate HV correction of his right foot. Weightbearing radiographic images of anteroposterior view at (**A**) preoperative period, (**B**) 1-month follow-up, (**C**) 3-month follow-up, (**D**) 12-month follow-up, and (**E**) last follow-up at 60 months after surgery, showing bone callus consolidation and remodeling, a stable and long-lasting radiographic correction

There were 41 (41.8%) patients with mild HV and 57 (58.2%) patients with moderate HV (Fig. 5). The mean preoperative HVA was 23.36 ± 5.87° and decreased to 14.67 ± 6.43° at the last follow-up with a mean correction of 8.69° (*p* < 0.0001). The mean IMA value decreased from 11.95 ± 2.60° preoperatively to 9.53 ± 2.83° at the last follow-up, with a mean correction of 2.42° (*p* < 0.0001). The mean preoperative DMAA was 9.88 ± 5.68° and 9.79 ± 7.88° at the last follow-up, with a mean correction of 0.09°. The median dislocation of the medial sesamoid was 1, both preoperatively and at the last follow-up (Table 1).

#### MIIND group

**Fig. 6:**
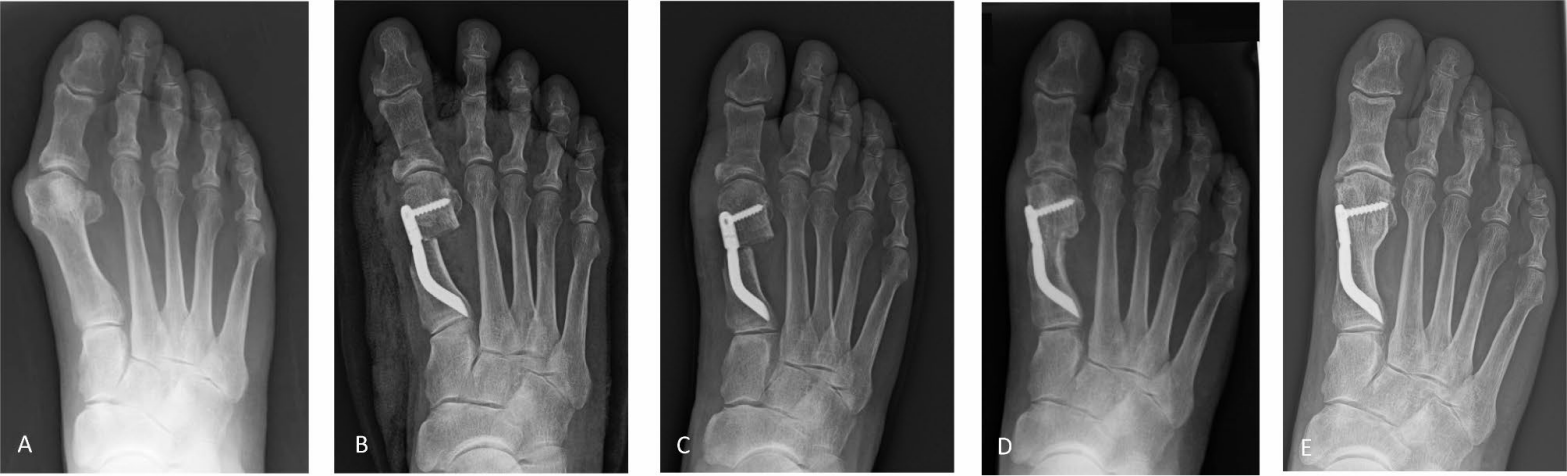
Case 2: A 58-year-old woman having undergone the Minimally Invasive Intramedullary Nail Device technique with Akin percutaneous osteotomy for severe HV correction of her right foot. Weightbearing radiographic images of anteroposterior view at (**A**) preoperative period, (**B**) 1-month follow-up, (**C**) 3-month follow-up, (**D**) 12-month follow-up, and (**E**) last follow-up at 60 months after surgery, showing bone callus consolidation and remodeling, a stable and long-lasting radiographic correction

In this group, there were 58 (59.2%) patients with moderate HV and 40 (40.8%) with severe HV (Table 1; Fig. 6). The mean HVA was 35.42 ± 9.57° preoperatively and 10.50 ± 8.41° at the last follow-up, with a mean correction of 24.92° (*p* < 0.0001). The mean IMA value decreased from 14.57 ± 3.12° preoperatively to 5.82 ± 3.24° at the last follow-up, with a mean correction of 8.75° (*p* < 0.0001).

The mean preoperative DMAA was 14.05 ± 6.02°, while it was 7.77 ± 5.96° at the last follow-up, with a mean correction of 6.28° (*p* < 0.0001). The median preoperative dislocation of the medial sesamoid was 3, while its value was 0 at the last follow-up.

#### PSM model

**Fig. 7:**
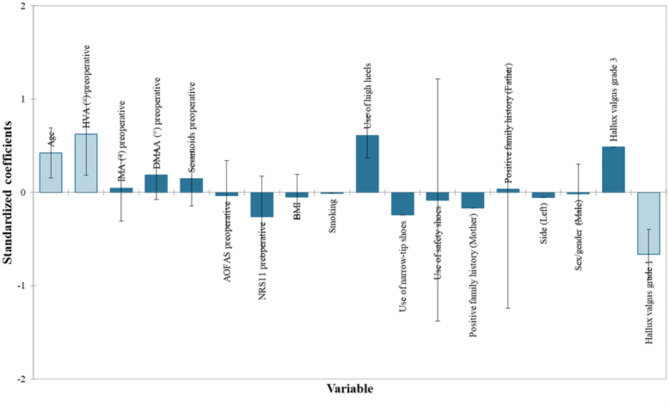
Impact of each variable on the PSM algorithm

The variables that impacted the allocation to a specific surgical technique (RIO versus MIIND) were age (OR 1.06 [95%CI 1.02–1.09], *p* = 0.002), preoperative HVA values (1.12 [95%CI 1.03–1.21], *p* = 0.005), and HV severity (OR 0.05 [95%CI 0.00-0.68] for grade 1 vs. grade 2, *p* = 0.024 (Table 2). In other words, older patients with greater preoperative HVA values, and more severe HV were more likely to be treated with MIIND (Fig. 7).

**Table 2:** Logistic regression model shedding light on the parameters associated with the type of surgery

Source	Unstandardized coefficients		Standardized coeffcients		Wald Chi-Square	Pr > Chi²	OR (95%CI)
	Value	Standard error	Value	Standard error			
-*2 Log(Likelihood) 66*,*024; R²(McFadden) 0.76; R²(Cox and Snell) 0.65; R²(Nagelkerke) 0.87; AIC 104.02; SBC 166.31; AUC 0.95*							
Intercept	-4.68	5.21			0.81	0.369	
Age	0.05	0.02	0.42	0.14	9.52	**0.002**	1.06 (1.02–1.09)
Sex/gender (male vs. female)	-0.07	0.85	-0.01	0.16	0.01	0.931	0.93 (0.18–4.93)
BMI	-0.02	0.06	-0.05	0.12	0.15	0.701	0.98 (0.87–1.10)
Smoking	-0.04	0.50	-0.01	0.12	0.01	0.931	0.96 (0.36–2.54)
HVA preoperative	0.11	0.04	0.62	0.22	7.72	**0.005**	1.12 (1.03–1.21)
IMA preoperative	0.03	0.10	0.05	0.18	0.07	0.799	1.03 (0.84–1.26)
DMAA preoperative	0.05	0.04	0.18	0.13	1.89	0.169	1.06 (0.98–1.14)
Sesamoids preoperative	0.24	0.25	0.15	0.15	0.96	0.328	1.28 (0.78–2.08)
AOFAS preoperative	-0.01	0.05	-0.04	0.19	0.03	0.856	0.99 (0.91–1.09)
NRS-11 preoperative	-0.36	0.31	-0.26	0.22	1.39	0.238	0.70 (0.38–1.27)
Narrow-tip shoes (no vs. yes)	-0.88	2.37	-0.24	0.65	0.14	0.709	0.413 (0.00-43.30)
High Heels (no vs. yes)	2.22	2.41	0.61	0.66	0.85	0.357	9.21 (0.08-1040.44)
Safety shoes (yes vs. no)	-0.63	1.22	-0.08	0.16	0.26	0.607	0.53 (0.05–5.84)
Family history– mother	-0.65	0.53	-0.17	0.14	1.50	0.221	0.52 (0.19–1.48)
Family history - father	0.28	1.07	0.03	0.12	0.07	0.793	1.32 (0.16–10.77)
Side (left vs. right)	-0.19	0.47	-0.05	0.13	0.17	0.685	0.83 (0.33–2.07)
Hallux valgus– grade 3 vs. 2	2.18	1.49	0.48	0.33	2.15	0.143	8.85 (0.48-163.51)
Hallux valgus– grade 1 vs. 2	-2.97	1.32	-0.67	0.30	5.08	**0.024**	0.05 (0.00-0.68)

BMI = Body Mass Index; HVA = hallux valgus angle; IMA = intermetatarsal angle; DMAA = distal metatarsal articular angle; AOFAS = American Orthopaedic Foot and Ankle Society score; NRS = Numeric Rating Scale

Propensity score values (as logit) for each observation are reported in Additional File 3, showing the matching between “treatment” and “control” observations along with their computed distances. The matching ensures comparability between the two groups for evaluating the effectiveness of RIO and MIIND surgical techniques.

### Comparison between the two surgical techniques (RIO vs. MIIND)

After implementing the PSM model, the two groups differed regarding IMA correction and HVA decrease. The former was significantly different between the two groups at three months (mean difference of 4.43 ± 3.54°, *p* < 0.0001), one year (mean difference of 4.44 ± 3.58°, *p* < 0.0001), and sixty months (mean difference of 4.49 ± 3.92°, *p* < 0.0001). The mean difference in HVA between the two groups at three months, one year, and sixty months was 5.68 ± 8.67° (*p* = 0.001), 4.36 ± 9.79° (*p* = 0.017), and 4.63 ± 9.84° (*p* = 0.015). In contrast, no differences could be found in DMAA correction. The mean difference in DMAA between the two groups at three months, one year, and sixty months resulted to be 2.81 ± 10.40° (*p* = 0.129), 2.57 ± 9.53° (*p* = 0.171), and 3.36 ± 10.17° (*p* = 0.084), respectively. Finally, concerning the correction of sesamoids, there was a significant difference between the two groups only at three months (*p* = 0.040) but not at 1 year (*p* = 0.277) or sixty months (*p* = 0.151) (Table 3).

**Table 3:** Comparison between the two surgical techniques

Parameter	RIO (*n* = 30)	MIIND (*n* = 30)	*P*-value
HVA (°) 3 months	15.44 ± 7.34	9.76 ± 6.26	***0.001***
IMA (°) 3 months	10.48 ± 2.97	6.06 ± 2.78	*< 0* ***.001***
DMAA (°) 3 months	11.19 ± 8.20	8.38 ± 5.69	*0.129*
HVA (°) 12 months	15.14 ± 7.08	10.78 ± 7.91	***0.017***
IMA (°) 12 months	10.35 ± 2.64	5.91 ± 2.86	*< 0* ***.001***
DMAA (°) 12 months	10.81 ± 7.22	8.25 ± 5.88	*0.171*
HVA (°) 60 months	15.59 ± 6.77	10.96 ± 8.63	***0.015***
IMA (°) 60 months	10.37 ± 2.86	5.88 ± 3.21	*< 0* ***.001***
DMAA (°) 60 months	11.75 ± 8.08	8.38 ± 6.46	*0.084*
Sesamoids 3 months	1	0	***0.040***
Sesamoids 12 months	1	0	*0.277*
Sesamoids 60 months	1	0	*0.151*
AOFAS 3 months	70.87 ± 8.41	72.50 ± 7.61	*0.428*
AOFAS 12 months	77.93 ± 12.5	81.93 ± 10.4	*0.262*
AOFAS 60 months	84.63 ± 14.4	89.20 ± 11.7	*0.232*
NRS-11 60 months	1.27 ± 1.36	1.13 ± 1.36	*0.792*
VAS 60 months	6.60 ± 2.39	7.67 ± 2.34	*0.091*

MIIND = Minimally Invasive Intramedullary Nail Device; RIO = Reverdin Ishan Osteotomy; HVA = hallux valgus angle; IMA = intermetatarsal angle; DMAA = distal metatarsal articular angle; AOFAS = American Orthopaedic Foot and Ankle Society score; NRS = Numeric Rating Scale; VAS = visual analogic scale

Data are reported as mean ± standard deviation

### Clinical functional outcomes

In the initial population, the mean preoperative AOFAS score was 50.29 ± 7.47 and increased to 86.52 ± 11.51 at the last follow-up (Table 1). The mean preoperative AOFAS score in the RIO group was 52.63 ± 6.53 and improved to 85.23 ± 13.02 at the last follow-up (*p* < 0.0001). In the MIIND group, the mean preoperative AOFAS score was 47.95 ± 7.64, and it increased over time, reaching 69.95 ± 7.43 at three months, 81.57 ± 8.55 at 12 months, and 87.80 ± 9.68 at the last follow-up (*p* < 0.0001). NRS-11 decreased from 6.41 ± 1.10 preoperatively to 1.23 ± 1.36 in the RIO group (*p* < 0.0001), while it decreased from 7.46 ± 1.28 to 1.31 ± 1.38 at 60 months in the MIIND group (*p* < 0.0001).

At the last follow-up, the mean VAS score for patient satisfaction was 6.84 ± 2.19 in the RIO group and 7.46 ± 2.13 in the MIIND group.

After the implementation of the PSM algorithm, no differences in AOFAS could be computed between the two groups at three months (mean difference 1.63 ± 10.58, *p* = 0.428), one year (mean difference of 4.00 ± 17.18, *p* = 0.262) and sixty months (mean difference of 4.57 ± 19.33, *p* = 0.232). Similarly, no differences in NRS-11 (mean difference of 0.13 ± 2.05, *p* = 0.792) or in patient satisfaction at 60 months could be detected (with a mean difference of 1.07 ± 3.56, *p* = 0.091) (Table 3).

### Complications

Major complications (13 patients, 6.63%) included 8 cases of recurrence and one case of severe stiffness (ROM < 30°) in the RIO group; 5 cases of recurrence were observed at the last follow-up in the MIIND group.

Minor complications (37 patients, 18.88%) included a slight loss of normal range of MTP joint motion (ROM 30°-74°) in 21 cases of the RIO group and 11 in the MIIND group, respectively. Furthermore, there were superficial wound infections in 4 patients in the MIIND group that were treated successfully with antibiotic therapy and one case of delayed wound healing because of portal burns during the RIO procedure.

## Discussion

Nowadays, several surgical techniques are used to treat HV, but the current literature does not provide consensus on which technique for the treatment of HV leads to the best outcomes [5, 6, 38].

While many studies have evaluated a specific percutaneous or MIS technique or have compared various MIS techniques with one another or with open techniques [4, 5, 20, 22, 39], to date, a comparison between RIO and MIIND techniques has only been performed in a small cohort (40 patients) with moderate HV, short follow-up and basic analysis [40].

In our series, patients treated by RIO had a reduction in HVA and IMA, comparing preoperative values with those at the last follow-up with a mean correction of 8.69° and 2.42°, respectively. A slight and marginal reduction in the DMAA was also obtained in this group. These findings appear comparable to studies on the same surgical technique [26, 40, 41]. Moreover, our results are in the range of correction found in the studies discussed in the review of Malagelada et al. [6]. Isham himself stated that the average reduction of the HVA and IMA is especially noted when the RIO procedure is associated with Akin osteotomy and lateral release, which contributes to the lateral movement of the first metatarsal axis and decreases the varus deformity [42]. For this last reason, combining Akin osteotomy during the most traditional procedures remains an attractive option also in open surgery [43]. The MIIND group also had reduced HVA and IMA values. Using the intramedullary nail device resulted in better HVA correction compared to other studies [40]. In the review performed by Jeyaseelan and Malagelada, the range of HVA correction comparing pre and postoperative values was 13.9–16.8° using the Endolog device and 12.9–20.8° using the Minimally Invasive/Percutaneous Chevron Akin (MICA or PECA), the percutaneous version of the MIIND techniques conceptually, but with a different fixation system (screws vs. nail) [20]. In our study, the satisfying IMA correction by MIIND was similar to the values found in other studies, whose potential for improving IMA was previously highlighted in the systematic review of Malagelada et al. [20].

The correction of DMAA, negligible in the RIO group through the closed wedge medial osteotomy, became an important correction in the MIIND group due to the concomitant operative derotation and the sizeable lateral displacement of the I- metatarsal head allowed by the device. At the same time, these two surgical steps promoted the reduction of the tibial sesamoid (TSP), while the internal fixation played a significative role in preventing the recurrence of valgism over time. In both groups, the postoperative values of the analysed angles improved compared to the preoperative ones, but the original correction tended to decrease several months after surgery while remaining within normal values.

The satisfactory results of our study were comparable to those of studies performed using both RIO and MIIND techniques compared to other percutaneous internal fixation techniques [44].

Di Giorgio et al., who compared RIO and MIIND techniques in 20 patients each, obtained excellent results in both groups but did not detect significant differences for HVA and IMA [40]. However, the number of patients was smaller, the follow-up shorter, and only patients with moderate HV were enrolled.

In our study, the MIIND technique provided more satisfactory correction of HVA and IMA than the RIO technique. This is because MIIND involves a complete translation of the MTH with internal fixation, providing a greater correction than the closed wedge medial osteotomy in severe HV [26]. The potential for improving IMA with MIIND was also highlighted in the review of Malagelada et al. [6].

Further, according to the authors, RIO appears to have the least potential for correcting both IMA and HVA angles compared to the other percutaneous and MIS techniques. However, these findings are not supported by evidence. Similar results were found by Lewis et al., who employed the PECA [45].

It has already been reported that the traditional Chevron osteotomy when compared with traditional open surgery, like the scarf procedure, had significantly more favourable postoperative outcomes in terms of HVA correction but not in terms of IMA. Distal chevron osteotomy provides greater HVA correction than scarf osteotomy, while proximal Chevron provides a larger IMA correction than distal chevron osteotomy [46, 47].

This study used a logistic regression model to find the surgery-associated variables. Age, preoperative HVA values and HVA severity were the variables that affected the allocation of a patient to a specific group. Older patients, with greater preoperative HVA values and thus more severe HV, were more likely to be treated with MIIND. A PSM model was applied to compare the two groups, reducing the bias and confounding factors and allowing a robust comparison between the two groups of 30 people each. Significant differences were observed between the two groups regarding HVA and IMA (at three months, one year and sixty months) and sesamoids (at three months), while no differences were found regarding DMAA. Therefore, angle reduction not only occurs early but is maintained over time, even at a significant follow-up of 60 months.

Regarding clinical outcomes, the AOFAS and NRS-11 scores improved after surgery in both groups. Similar results were observed by other authors [6, 20]. In the MIIND group, improvement in the AOFAS score was found at the last follow-up, with lower improvement values than in other studies, which had shorter follow-ups [6, 20]. Significantly lower preoperative levels than in our study can explain better AOFAS score improvement. However, after implementing the PSM algorithm, no differences in AOFAS scores were computed between the two groups at three months, one year and sixty months. Similarly, Di Giorgio et al. did not find significant differences in the clinical scores [40].

No differences could be detected regarding VAS satisfaction at 60 months comparing the two groups [40] in agreement with our study.

A meta-analysis compared MIS vs. OPEN techniques, highlighting the superiority of MIS techniques in the early postoperative period (shorter surgery time, a more cosmetic scar, a higher satisfaction rate, and a faster recovery time) [4]. Although MIS for HV is considered safe and effective, with radiological, functional, and clinical results comparable to open procedures, there is currently insufficient literature evidence to recommend MIS over open procedures or favour one MIS technique over another.

Our study aligns with the literature that recommends using percutaneous or MIS techniques without internal fixation only for mild HV and suggests techniques involving internal fixation for severe HV [22, 45, 48, 49]. Percutaneous techniques such as RIO applied to severe radiological deformities may not give the same results, mainly because they lack internal fixation that maintains the correction [50].

In our groups, minor complications (18.8%) were prevalent and resolved with medical therapy. The value was slightly lower than that reported in the literature (21.42% vs. 1% in the RIO group; 11.22% vs. 1–2% in the MIIND group) [6, 20]. Major complications occurred in 8.16% of patients treated by RIO and in 5.10% of the MIIND group. These were in line with those found by other authors (5–11% major complications in the RIO group and 2–3% in the MIIND one, respectively) [6, 20], and they were resolved by revision surgery, yielding satisfying results after accurate preoperative planning and identification of causes of failure [51].

The strengths of our study include (1) it is the first that analyses the long-term radiographic and clinical outcomes of a large patient cohort by performing RIO or MIIND techniques in association with Akin osteotomy, (2) both techniques were used to correct a painful HV only on one foot without additional procedures on the lateral rays, (3) the standardisation of patient operations and postoperative protocol, (4) the evaluation of the clinical and radiographic outcomes carried out separately by blinded investigators and (5) the application of the PSM analysis model, which enabled a robust comparison of the two groups.

The main limitations of our study include (1) a single-centre study, (2) the retrospective nature of the study, (3) the relatively small sample size and (4) the use of the AOFAS score, only partially validated [52], having a single question related to pain and correlating poorly with the SF-36 in patients with foot complaints [52, 53]. However, AOFAS remains the most widespread health measurement in foot and ankle clinical practice, allowing the formulation of valid conclusions related to foot and ankle quality-of-life issues [54, 55].

## Conclusions

Our study demonstrates the efficacy of the RIO and MIIND techniques assigned according to the severity of HV. We conclude that patients with higher HVA and moderate-to-severe HV should be treated with the MIIND technique, while subjects with mild-to-moderate deformity should undergo RIO. Overall, radiographic and clinical outcomes improved in patients treated by both methods. However, patients treated with MIIND had better angular values at all follow-ups compared to RIO, while no differences were observed regarding DMAA correction, AOFAS scores, NRS-11 and VAS satisfaction.

Further studies are needed to confirm our data, such as randomised controlled trials with appropriate sample sizes, validated outcome measures, blinded assessors and long-term follow-up to determine the efficacy of MIS techniques.

## Electronic supplementary material

Below is the link to the electronic supplementary material.

Supplementary Material 1

Supplementary Material 2

Supplementary Material 3

## Data Availability

No datasets were generated or analysed during the current study.
