# Supplementary material for: Comparative analysis between Reverdin-Isham Osteotomy (RIO) and minimally invasive intramedullary nail device (MIIND) in association with AKIN osteotomy for Hallux valgus correction
Source: J Orthop Surg Res. 2025 Feb 20;20:185. doi: 10.1186/s13018-025-05569-7 (PMC11844019; doi:10.1186/s13018-025-05569-7)
Supplement: Supplementary file 1 — Supplementary Material 1 [file 13018_2025_5569_MOESM1_ESM.docx]

**Comparative Analysis Between Reverdin-Isham Osteotomy (RIO) and Minimally Invasive Intramedullary Nail Device (MIIND) in association with AKIN osteotomy for Hallux Valgus Correction.**

**Additional File 1. Hallux valgus radiographic classification based on severity of deformity.**

| **Severity of Deformity** | **IMA** | **HVA** | **TSP subluxation** |
| --- | --- | --- | --- |
| Normal | < 10° | ≤ 15° | No displacement (grade 0) |
| Mild | 10 -11° | 16 - 19° | < 50 % (grade 1) |
| Moderate | 12-15° | 20 - 40° | 50 – 75 % (grade 2) |
| Severe | ≥ 16° | > 40° | > 75 % (grade 3) |

IMA= intermetatarsal angle; HVA= hallux valgus angle; TSP=tibial sesamoid position.
