# Supplementary material for: Comparative analysis between Reverdin-Isham Osteotomy (RIO) and minimally invasive intramedullary nail device (MIIND) in association with AKIN osteotomy for Hallux valgus correction
Source: J Orthop Surg Res. 2025 Feb 20;20:185. doi: 10.1186/s13018-025-05569-7 (PMC11844019; doi:10.1186/s13018-025-05569-7)
Supplement: Supplementary file 2 — Supplementary Material 2 [file 13018_2025_5569_MOESM2_ESM.docx]

**Comparative Analysis Between Reverdin-Isham Osteotomy (RIO) and Minimally Invasive Intramedullary Nail Device (MIIND) in association with AKIN osteotomy for Hallux Valgus Correction.**

**Additional File 2**

A propensity score analysis (PSA) was implemented to compare the two techniques [1]. PSA was carried out employing the commercial software XLSTAT (version for Windows OS, Lumivero) [2]. The propensity scores were estimated using a logistic regression model in which the surgical treatment (RIO vs MIIND) status was regressed on observed characteristics (covariates and factors). These scores were used to adjust an ordinary least-squares regression mixed model for repeated measures data, which enabled the computation of the estimates of the effect of the surgical technique, the time-points (3, 12, and 60 months), and their interactions. Along with the scores, the regression models were adjusted for all confounders (covariates and factors) previously used in the PSA.

Results of the analysis are reported in Tables 2.1 and 2.2.

**Table 2.1 Impact of the surgical technique, time-point, and their interaction on the study outcomes.**

| **Parameters** | **F-value** | ***p-value*** |
| --- | --- | --- |
| **HVA** | | |
| Surgical technique | 8.27 | *0.004* |
| Time | 503.23 | *< 0.001* |
| Surgery ✻ Time | 109.38 | *< 0.001* |
| **IMA** | | |
| Surgical technique | 33.62 | *< 0.001* |
| Time | 411.12 | *< 0.001* |
| Surgery ✻ Time | 130.51 | *< 0.001* |
| **DMAA** | | |
| Surgical technique | 7.33 | *0.007* |
| Time | 30.04 | *< 0.001* |
| Surgery ✻ Time | 24.17 | *< 0.001* |
| **Sesamoids** | | |
| Surgical technique | 5.21 | *0.024* |
| Time | 159.95 | *< 0.001* |
| Surgery ✻ Time | 51.49 | *< 0.001* |
| **AOFAS** | | |
| Surgical technique | 0.705 | *0.402* |
| Time | 1380.153 | *< 0.001* |
| Surgery ✻ Time | 16.032 | *< 0.001* |

HVA= hallux valgus angle; IMA= intermetatarsal angle; DMAA= distal metatarsal articular angle; AOFAS= American Orthopaedic Foot and Ankle Society score.

**Table 2.2 Estimates of the propensity score-adjusted ordinary least squares regression model comparing RIO and MIIND techniques over time (at 3, 12, and 60 months).**

| **Parameters** | **Estimate** | **SE** | **t-value** | ***p-value*** |
| --- | --- | --- | --- | --- |
| **HVA** | | | | |
| RIO vs MIIND | 3.74 | 1.300 | 2.88 | *0.004* |
| 3 mo | -17.52 | 0.541 | -32.38 | *< 0.001* |
| 12 mo | -17.13 | 0.541 | -31.67 | *< 0.001* |
| 60 mo | -16.80 | 0.541 | -31.06 | *< 0.001* |
| RIO ✻ 3 mo | 16.20 | 1.082 | 14.97 | *< 0.001* |
| RIO ✻ 12 mo | 15.53 | 1.082 | 14.35 | *< 0.001* |
| RIO ✻ 60 mo | 16.24 | 1.082 | 15.01 | *< 0.001* |
| **IMA** | | | | |
| RIO vs MIIND | 3.27 | 0.564 | 5.80 | *< 0.001* |
| 3 mo | -5.23 | 0.191 | -27.31 | *< 0.001* |
| 12 mo | -5.61 | 0.191 | -29.31 | *< 0.001* |
| 60 mo | -5.59 | 0.191 | -29.22 | *< 0.001* |
| RIO ✻ 3 mo | 6.03 | 0.383 | 15.75 | *< 0.001* |
| RIO ✻ 12 mo | 6.18 | 0.383 | 16.14 | *< 0.001* |
| RIO ✻ 60 mo | 6.33 | 0.383 | 16.54 | *< 0.001* |
| **DMAA** | | | | |
| RIO vs MIIND | 3.12 | 1.154 | 2.71 | *0.007* |
| 3 mo | -3.31 | 0.442 | -7.48 | *< 0.001* |
| 12 mo | -3.70 | 0.442 | -8.38 | *< 0.001* |
| 60 mo | -3.18 | 0.442 | -7.20 | *< 0.001* |
| RIO ✻ 3 mo | 6.62 | 0.884 | 7.49 | *< 0.001* |
| RIO ✻ 12 mo | 5.37 | 0.884 | 6.08 | *< 0.001* |
| RIO ✻ 60 mo | 6.18 | 0.884 | 6.99 | *< 0.001* |
| **Sesamoids** | | | | |
| RIO vs MIIND | 0.326 | 0.1427 | 2.28 | *0.024* |
| 3 mo | -1.158 | 0.0648 | -17.87 | *< 0.001* |
| 12 mo | -1.179 | 0.0648 | -18.18 | *< 0.001* |
| 60 mo | -1.140 | 0.0649 | -17.57 | *< 0.001* |
| RIO ✻ 3 mo | 1.235 | 0.1296 | 9.53 | *< 0.001* |
| RIO ✻ 12 mo | 1.337 | 0.1296 | 10.31 | *< 0.001* |
| RIO ✻ 60 mo | 1.362 | 0.1298 | 10.49 | *< 0.001* |
| **AOFAS** | | | | |
| RIO vs MIIND | -1.51 | 1.796 | -0.840 | *0.402* |
| 3 mo | 20.31 | 0.604 | 33.625 | *< 0.001* |
| 12 mo | 30.14 | 0.604 | 49.906 | *< 0.001* |
| 60 mo | 36.22 | 0.604 | 59.985 | *< 0.001* |
| RIO ✻ 3 mo | -3.39 | 1.208 | -2.805 | *0.005* |
| RIO ✻ 12 mo | -6.97 | 1.208 | -5.770 | *< 0.001* |
| RIO ✻ 60 mo | -7.24 | 1.208 | -5.998 | *< 0.001* |

HVA= hallux valgus angle; RIO=Reverdin Ishan Osteotomy; MIIND= Minimally Invasive Intramedullary Nail Device; IMA= intermetatarsal angle; DMAA= distal metatarsal articular angle; AOFAS= American Orthopaedic Foot and Ankle Society score.

1. Kane LT, Fang T, Galetta MS, et al. Propensity Score Matching: A Statistical Method. Clin Spine Surg. 2020;33(3):120-122. doi:10.1097/BSD.0000000000000932
2. Spiel C, Lapka D, Gradinger P, et al. A Euclidean distance-based matching procedure for nonrandomized comparison studies. Eur Psychol. 2008;13(3):180-187. doi:10.1027/1016-9040.13.3.187
