# Supplementary material for: Comparative analysis between Reverdin-Isham Osteotomy (RIO) and minimally invasive intramedullary nail device (MIIND) in association with AKIN osteotomy for Hallux valgus correction
Source: J Orthop Surg Res. 2025 Feb 20;20:185. doi: 10.1186/s13018-025-05569-7 (PMC11844019; doi:10.1186/s13018-025-05569-7)
Supplement: Supplementary file 3 — Supplementary Material 3 [file 13018_2025_5569_MOESM3_ESM.docx]

**Comparative Analysis Between Reverdin-Isham Osteotomy (RIO) and Minimally Invasive Intramedullary Nail Device (MIIND) in association with AKIN osteotomy for Hallux Valgus Correction.**

**Additional File 3. Details of the matched observations between the treatment and control groups based on their logit (propensity score).**

| **Treatment** | **Logit (Propensity score)** | **Control** | **Logit (Propensity score)** | **Distances** |
| --- | --- | --- | --- | --- |
| Obs1 | -0.098 | Obs102 | -0.233 | 0.134 |
| Obs4 | -0.073 | Obs167 | -0.082 | 0.009 |
| Obs5 | 0.293 | Obs 99 | 0.224 | 0.068 |
| Obs6 | -0.514 | Obs151 | -0.500 | 0.015 |
| Obs7 | -0.068 | Obs116 | -0.113 | 0.045 |
| Obs10 | -0.046 | Obs120 | -0.199 | 0.154 |
| Obs11 | 0.891 | Obs143 | 0.867 | 0.024 |
| Obs14 | 0.034 | Obs115 | -0.144 | 0.179 |
| Obs16 | -0.140 | Obs148 | -0.228 | 0.088 |
| Obs17 | -0.302 | Obs142 | -0.304 | 0.002 |
| Obs23 | -0.079 | Obs147 | -0.081 | 0.001 |
| Obs28 | 0.124 | Obs118 | -0.014 | 0.138 |
| Obs31 | -0.235 | Obs119 | -0.276 | 0.040 |
| Obs41 | 0.836 | Obs113 | 0.820 | 0.016 |
| Obs42 | 0.152 | Obs176 | 0.072 | 0.080 |
| Obs48 | 0.188 | Obs136 | 0.097 | 0.091 |
| Obs50 | -0.617 | Obs130 | -0.635 | 0.018 |
| Obs52 | 0.318 | Obs114 | 0.315 | 0.003 |
| Obs54 | 0.249 | Obs166 | 0.213 | 0.035 |
| Obs58 | -0.702 | Obs132 | -0.652 | 0.051 |
| Obs59 | 0.172 | Obs131 | 0.044 | 0.129 |
| Obs60 | -0.124 | Obs170 | -0.150 | 0.026 |
| Obs66 | 0.702 | Obs173 | 0.705 | 0.004 |
| Obs79 | 0.395 | Obs146 | 0.349 | 0.046 |
| Obs85 | -0.200 | Obs103 | -0.208 | 0.009 |
| Obs89 | -0.477 | Obs182 | -0.473 | 0.004 |
| Obs90 | -0.092 | Obs172 | -0.123 | 0.031 |
| Obs92 | 0.287 | Obs126 | 0.272 | 0.015 |
| Obs95 | 0.364 | Obs158 | 0.361 | 0.003 |
| Obs97 | 0.034 | Obs139 | -0.106 | 0.140 |
